# Belief Propagation Optimization for Lossy Compression Based on Gaussian Source

**DOI:** 10.3390/s23218805

**Published:** 2023-10-29

**Authors:** Huan Deng, Dan Song, Zhiping Xu, Yanglong Sun, Lin Wang

**Affiliations:** 1 Navigation Collage, Jimei University, Xiamen 361021, China; 2 Key Laboratory of Underwater Acoustic Communication and Marine Information Technology (Xiamen University), Ministry Education, Xiamen 361005, China; 3 School of Ocean Information Engineering, Jimei University, Xiamen 361021, China; 4 Department of Information and Communication Engineering, Xiamen University, Xiamen 361005, China; 5 Hangzhou Microimage Software Co., Ltd., Hangzhou 310051, China

**Keywords:** lossy source coding, P-LDPC code, trapping set, BP algorithm

## Abstract

In the Internet of Things, sensor nodes collect environmental information and utilize lossy compression for saving storage space. To achieve this objective, high-efficiency compression of the continuous source should be studied. Different from existing schemes, lossy source coding is implemented based on the duality principle in this work. Referring to the duality principle between the lossy source coding and the channel decoding, the belief propagation (BP) algorithm is introduced to realize lossy compression based on a Gaussian source. In the BP algorithm, the log-likelihood ratios (LLRs) are iterated, and their iteration paths follow the connecting relation between the check nodes and the variable nodes in the protograph low-density parity-check (P-LDPC) code. During LLR iterations, the trapping set is the main factor that influences compression performance. We propose the optimized BP algorithms to weaken the impact of trapping sets. The simulation results indicate that the optimized BP algorithms obtain better distortion–rate performance.

## 1. Introduction

In the rapid development of Internet of Things (IoT) technology, some new technologies have been introduced in recent works, such as energy harvesting [1], backscatter [2], network virtualization [3], radar-communication [4], and a new battery [5]. These high-tech methods effectively promote the development of the wireless communication system of IoT. More importantly, high-efficiency and low-cost requirements are two objectives in the physical layer of the communication system. In IoT, the sensor nodes collect the environment data and send them to the receiving nodes. Generally, the collected data are modeled as the Gaussian source. Here are two useful techniques for gaining the two objectives in the physical layer. First, to achieve high efficiency, lossy source coding [6] is implemented to compress the source data for reducing source redundancy [7,8,9]. Second, to obtain low cost, the existing channel decoding algorithm is reused to realize the lossy source coding according to the duality principle [10,11,12].

The low-density parity-check (LDPC) code performs excellently in the fields of channel coding and source coding, especially in joint source channel coding [13,14]. Moreover, their code design and decoding improvement provide better system performance, which demonstrates that the LDPC codes have good coding property. However, most of the existing works focus on realizing lossless compression of the binary source, which is difficult to promote to the continuous source, and its compression efficiency needs to be improved. Furthermore, there is little research paying attention to the design of lossy compression schemes that are more appropriate for practical applications. Therefore, it is critical to study lossy compression of the continuous source based on LDPC code with lower complexity and higher performance.

Recently, protograph-LDPC (P-LDPC) code [15] was introduced to compress a Gaussian source via lossy source coding in [16] since P-LDPC is a simplified structure of the LDPC code. Furthermore, the traditional channel decoding algorithm, i.e., the belief propagation (BP) algorithm, completes the quantization of lossy source coding to compress a Gaussian source. In [17], a multilevel coding (MLC) structure with a binary mapping scheme was designed to compress a Gaussian source. These two works have some differences. First, ref. [16] is a concatenated coding system and ref. [17] is an MLC system. Second, ref. [16] directly compresses a float into one bit, whereas [17] maps a float into a binary string.

In summary, refs. [16,17] are two different kinds of lossy source coding systems. However, it was found that their compression performance could not approach the rate–distortion limit. In this work, we focus on optimizing the MLC structure and aim to decrease the number of trapping sets in the BP algorithm. Technically, the BP algorithm is an efficient method for realizing the lossy source coding [18,19,20,21,22] and the channel decoding [23,24,25,26,27,28] simultaneously, where the trapping sets influence the iteration performance and derive a series of bit errors both in the source and the channel coding [29,30]. In this case, there are some works concentrating on improving the BP algorithm.

**Figure 1:**
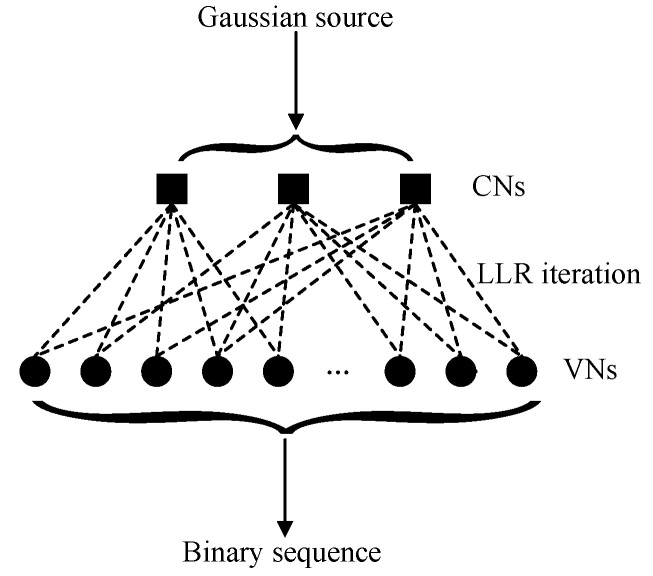
The compression principle of the BP algorithm based on P-LDPC code for a Gaussian source.

In the BP algorithm, log-likelihood ratios (LLRs) are iterated based on the connecting relation between the check nodes (CNs) and the variable nodes (VNs) in the P-LDPC code [31,32,33]. The compression principle is shown in detail inn Figure 1. If the P-LDPC code is determined, the connection relation between CNs and VNs is fixed. Each Gaussian variable is input as the initial LLR, and it is iterated following the connection relation to obtain the binary sequence. During the iteration, the trapping sets will stop the LLR convergence to find the optimal codeword [34]. To resolve this problem, the multi-stage BP (MSBP) [35], the backtracking BP (BBP) [36], and the two-stage BP (TSBP) [29] algorithms are proposed to weaken the influence of the trapping sets in the BP algorithms. These three algorithms are optimized from reducing the number of trapping sets to obtain good performance. In the MSBP algorithm, the trapping set is eliminated by setting zero to correct the target node, which improves the performance in high signal-to-noise ratio regions. The DBBP algorithm is proposed to roughly locate the trapping sets, so that the selection accuracy of the target nodes can be enhanced. In addition, two kinds of target nodes can be selected by the TSBP algorithm without increasing the complexity and reducing the reliability, which further improves the performance.

Generally, the aforementioned algorithms, including the MSBP, the BBP, and the TSBP, are implemented for channel decoding functions. According to the duality principle, these three algorithms are introduced to realize the lossy source coding in this work. An optimized MLC system is considered to compress the Gaussian source. Each employed algorithm is analyzed with its advantage in the lossy source coding system. Furthermore, the optimized MSBP (OMSBP) and the optimized TSBP (OTSBP) algorithms are proposed to improve the rate–distortion performance in the lossy source coding system.

Overall, two contributions are summarized as follows:

(1) The MLC structure is improved to realize the lossy compression of the Gaussian source with high efficiency. The improved MLC structure is a typical communication scheme applied in IoT, which will not only promote the distributed source coding, but also the cascading system will be ameliorated.

(2) The BP algorithms are optimized to obtain coding gains for the lossy source coding system with low complexity. It should be noted that the optimal methods are different, and their common objective is to reduce the number of trapping sets. The optimized BP algorithms can be further utilized as the channel decoding schemes according to the duality principle.

The rest of this paper is arranged as follows. Section 2 introduces the system model. Section 3 presents the principles and characteristics of the MSBP, the BBP, and the TSBP algorithms, while their optimizations are shown correspondingly. Section 4 gives some system simulation results to demonstrate the effectiveness of the optimizations. Section 5 concludes this paper.

## 2. System Model

**Figure 2:**
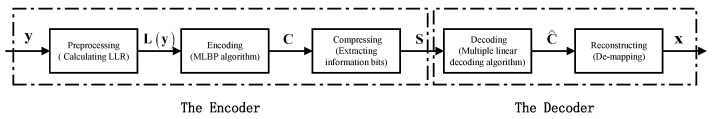
The lossy compression system based on the MLC structure for the Gaussian source.

The lossy compression system based on the MLC structure for the Gaussian source is shown in Figure 2. In the encoder, there are three modules, including the preprocessing, the encoding, and the compressing. The source y is a memoryless Gaussian sequence signified as $y=\{y_{0},\cdots ,y_{n-1}\}$, where $y_{n-1}\in R$, n∈N, R is the set of real numbers, and N is the set of natural numbers. The source sequence y is preprocessed, and its corresponding log-likelihood ratio (LLR) is calculated as $L\left(y\right)$. The encoding module employs the multilevel BP (MLBP) algorithm, which changes the LLR into bit. C is the encoded binary sequence. After that, C is compressed as the short binary sequence S for transmission. In the decoder, the transmission sequence S is decoded as $\hat{C}$ by using the multiple linear decoding algorithm [17]. Finally, $\hat{C}$ is reconstructed as the receiving source X by demapping.

It is found that the original MLBP algorithm has incorrect convergence during the iteration process. The main reason is that the LLR iterations will fall into the trapping set, which occurs the error floor. To solve this problem, the MLBP algorithm is optimized in this paper.

## 3. Optimization of BP Algorithms

### 3.1. OMSBP Algorithm

In [35], the MSBP algorithm is proposed as an optimized BP algorithm and aims to solve the trapping set problem. The optimization principle is to reduce the influence of unreliable information that comes from the iteration algorithms without locating the trapping sets. In the BP algorithm, the erroneous nodes can be divided into three types, namely, the unstable, the stable, and the oscillatory, where the oscillatory nodes account for the majority. The MSBP algorithm mainly optimizes the performance by addressing stable and oscillatory nodes, and it proposes a universal selection method for these two target nodes.

The main process of the original MSBP algorithm is stated as follows. First, the BP algorithm is applied to realize the initialized encoding. Then, the number of the CNs is determined. If this number satisfies the maximum coding stage, the encoding is terminated; otherwise, move to the next step. During the coding stage, two classes of indicators are selected as two objective VNs. These two classes of VNs are postprocessed, and the iteration is skipped to the previous step for implementing the next encoding stage.

The two classes of indicators are signified as

- $N_{sc}\left(j\right)$: The number of symbol changes of LLR in the *j*-th VN, i.e., the number of node information symbol changes.
- $N_{sd}\left(j\right)$: The different times of the symbol between the LLR in the *j*-th VN and the input LLR, i.e., the different times of node information symbol.

In the MSBP algorithm, the VNs are arranged in descending order according to $N_{sc}\left(j\right)$ or $N_{sd}\left(j\right)$; then, the first $N_{select}$ nodes are selected as the target nodes, where $N_{select}$ indicates the number of preset target nodes.

In this paper, we find that if the selected VNs are reliable, the ratio of the correct information will decline in the iteration. This is contradictory to the correct encoding process. To repair this bug, a new postprocessing method is proposed to minimize the loss of correct information when the corresponding nodes are selected with errors. Here, the postprocessing is modified as follows:

(1) $$L_{in,i}{\left(y_{j}\right)}^{\left(s+1\right)}=\frac{1}{2}\left(L_{in,i}{\left(y_{j}\right)}^{\left(s\right)}+L_{out,i}{\left(y_{j}\right)}^{\left(s\right)}\right)$$

where $L_{in,i}{\left(y_{j}\right)}^{\left(s\right)}$ and $L_{out,i}{\left(y_{j}\right)}^{\left(s\right)}$ represent the LLRs of the *j*-th VN at the *i*-th level before and after the *s*-th encoding stage, respectively. The values of *s* are arranged from 0 to S−1, where *S* is the total stage number.

### 3.2. DBBP Algorithm

From the aforementioned contents, it can be seen that the existing two multi-stage algorithms may have erroneous LLR observations on confirming the unreliable VNs. In addition, their batch-processing methods cannot guarantee the correctness of each objective VN. To resolve the low credibility, the BBP algorithm performs a rough localization on the main trapping set, according to the degrees that do not satisfy the CNs. Furthermore, the target nodes are processed with some appropriate methods to determine their correctness. In this case, the BBP algorithm achieves higher accuracy than the MSBP algorithm.

In detail, the BBP algorithm is shown in Algorithm 1. Different from the MSBP algorithm, the BBP will select the minimum set of CNs that are unsatisfied with the initialized encoding until the maximum iteration. After that, the BP principle is used as the backtracking encoding. Here, the LLR of the VN associated with the selected CN is flipped. In Algorithm 1, $H_{i}$ and $L_{in,i}\left(y\right)$ denote the check matrix and the input LLR sequence of the *i*-th level, respectively. $\Omega_{0}$ represents the minimum set of the unsatisfied CNs in the first encoding process, and $N\left(\Omega_{0}\right)$ is the set of VNs associated with $\Omega_{0}$. The parameter $u_{0}$ represents the output codeword corresponding to $\Omega_{0}$.

However, the BBP algorithm cannot always correct all errors, and it still has the risk of falling into a new trapping set. To improve these problems, the double-backtracking belief propagation (DBBP) algorithm is proposed. The main difference is that the DBBP algorithm can be simply described as if the first backtracking encoding fails, then the second backtracking is performed with $\Omega_{0}$ and $N\left(\Omega_{0}\right)$. Therefore, the DBBP algorithm has more of a chance to successfully encode and has a lower risk of falling into the new trapping set.
**Algorithm 1** BBP algorithm**Input **y, $H_{i}$, $L_{in,i}\left(y\right)$, $\Omega_{0}$, $N\left(\Omega_{0}\right)$ and $u_{0}$**Output:** The encoded bit sequence for each level: $C_{out}$ 1:/*Initializing: From Initial Encoding*/ 2:$L\left(y\right)=\left[L\left(y_{0}\right),L\left(y_{1}\right),\cdots ,L\left(y_{n_{v}-1}\right)\right]$: LLR intermediate vector $L\left(y\right)\leftarrow L_{in,i}\left(y\right)$ 3:η: the largest possible positive LLR 4:/*First Backtracking*/ 5:**for **$v\in N\left(\Omega_{0}\right)$ **do** 6:    $L\left(y\right)\leftarrow L_{in,i}\left(y\right)$ 7:    $L\left(y_{v}\right)\leftarrow -\chi \left(u_{m,v}\right)\cdot \eta$ 8:    re-encode using $L\left(y\right)$ as the input vector 9:    **if** re-decoding is successful **then**10:       stop and exit11:   **end if**12:**end for**

### 3.3. OTSBP Algorithm

The BBP algorithm is a kind of trial-and-error backtracking, which only has a coarse location on the trapping set. Technically, this algorithm has high complexity since it attempts to find the intersection between the associated VNs and the trapping set by tracing the unsatisfied CNs in several times. The TSBP algorithm is an improvement of the BBP. A criterion is designed to distinguish the two types of VNs with a high probability of incorrectly and correctly coding, in which the erroneous node information is flipped and the correct node information is multiplied. This is a high-efficiency way to increase the selection accuracy and the iteration performance. In addition, the termination criterion is introduced to avoid unnecessary iterations for achieving the lower complexity. Within the total iterations, the first stage will be terminated when the number of unsatisfied CNs are unchanged.

Similarly, the TSBP algorithm is divided into two stages, as shown in Algorithm 2. In the first stage, the initial encoding is started, and it can be terminated by the stop criterion. In the second stage, if the initial encoding fails, two types of target VNs will be selected according to their output LLRs. Furthermore, their input LLR information will be corrected by using different methods based on the type of target nodes. Then, the iteration moves to the second stage. Here, the selection criteria of two types of target VNs are described as follows.

- The first type of VNs:
  (2a) $$\begin{array}{cc} \; & sign\left(L_{in,i}\left(y_{j}\right)\right)\neq sign\left(L_{out,i}^{\left(1\right)}\left(y_{j}\right)\right) \end{array}$$
  (2b) $$\begin{array}{c} and\;|L_{in,i}\left(y_{j}\right)\left|\;>\;\right|L_{out,i}^{\left(1\right)}\left(y_{j}\right)| \end{array}$$
  (2c) $$\begin{array}{cc} \; & and\;|L_{in,i}\left(y_{j}\right)\left|\;+\;\right|L_{out,i}^{\left(1\right)}\left(y_{j}\right)|\;>\;\alpha . \end{array}$$

- The second type of VNs:
  (3a) $$\begin{array}{cc} \; & sign\left(L_{in,i}\left(y_{j}\right)\right)=sign\left(L_{out,i}^{\left(1\right)}\left(y_{j}\right)\right) \end{array}$$
  (3b) $$\begin{array}{c} and\;|L_{in,i}\left(y_{j}\right)\left|\;>\;\right|L_{out,i}^{\left(1\right)}\left(y_{j}\right)|. \end{array}$$

Here, $L_{in,i}\left(y_{j}\right)$ and $L_{out,i}^{\left(1\right)}\left(y_{j}\right)$ are input and output LLRs of the *j*-th VN in the first stage encoding at the *i*-th level, respectively. α is the span ceiling, and it is set to 3, generally. β is the multiplier factor and β=1.25.
**Algorithm 2** TSBP algorithm**Input:** y, $H_{i}$, $L_{out,i}^{\left(1\right)}\left(y\right)$ and *L***Output:** The encoded bit sequence for each level: $C_{out}$ 1:/*Initializing: From Initial Encoding*/ 2:$L\left(y\right)=\left[L\left(y_{0}\right),L\left(y_{1}\right),\cdots ,L\left(y_{n_{v}-1}\right)\right]$: intermediate vector $L\left(y\right)\leftarrow L_{out,i}^{\left(1\right)}\left(y\right)$ 3:/*Pre-processing for Type I Nodes*/ 4:**for **$a_{j}\in V_{1}$ **do** 5:    $L\left(y_{a_{j}}\right)=\beta \cdot L_{out,i}^{\left(1\right)}\left(y_{a_{j}}\right)j=0,1,2,\ldots ,n_{1}-1$ 6:**end for** 7:/*Flipping Type II Nodes and Re-encoding*/ 8:**for **$b_{j}\in V_{2}$ **do** 9:    $d\left(b_{j}\right)=\frac{|L_{out,i}^{\left(1\right)}\left(y_{b_{j}}\right)-L_{in,i}\left(y_{b_{j}}\right)|}{L_{in,i}\left(y_{b_{j}}\right)j}=0,1,2,\ldots ,n_{2}-1$10:**end for**11:**for **l=1⋯L **do**12:    Find the node $b_{j}$, which subjects to $d\left(b_{j}\right)=max\left(d\left(b_{0}\right),\cdots ,d\left(b_{n_{2}-1}\right)\right)$13:    Flip the LLR of node $b_{j}$: $L\left(y_{b_{j}}\right)=-\beta \cdot L_{out,i}^{\left(1\right)}\left(y_{b_{j}}\right)$14:    re-encode with the updated LLRs $L\left(y\right)$ and output $L_{de}\left(y\right)$15:    Make hard decision by $L_{de}\left(y\right)$ and calculate the number of unsatisfied check nodes $N_{u}$16:    **if** $N_{u}=0$ **then**17:        $L_{out,i}^{\left(2\right)}\left(y\right)=L_{de}\left(y\right)$18:        Output the decoded word $C_{out}$, then go to the end19:    **else**20:        Delete $d\left(b_{j}\right)$ from d21:        $L\left(y\right)=L_{de}\left(y\right)$22:    **end if**23:**end for**24:$L_{out,i}^{\left(2\right)}\left(y\right)=L_{de}\left(y\right)$25:Make hard decision by $L_{out,i}^{\left(2\right)}\left(y\right)$ and output the decoded word $C_{out}$

From the above two formulas, it can be seen that the first-type target nodes have the following characteristics: the symbols of the output and input LLRs are opposite and they have a large difference, i.e., the absolute values of the input LLRs are significantly greater than the output LLRs. Therefore, it can be considered that the output LLRs are affected by the incorrect interference from the input LLRs. However, the output LLRs will be corrected after the first stage. On the contrary, the second-type target nodes have the following characteristics: there are fewer differences between the output and input LLRs. The erroneous nodes are regarded as the ones disturbed by the input information, which are not corrected in the first stage. In the TSBP algorithm, the first type of nodes are considered as correct, whose LLRs are amplified; furthermore, the second type of nodes are considered as wrong, whose LLRs are flipped.

In Algorithm 2, $L_{out,i}^{\left(1\right)}\left(y\right)$ represents the output LLR sequence in the initial encoding at the *i*-th level, and *L* is the number of the second type of nodes to be flipped in re-encoding. $V_{1}$, $n_{1}$, $V_{2}$, and $n_{2}$ are the sets and the numbers of the first and the second type of VNs, respectively. From the eighth to the thirteenth line, the flipping order of the second type of nodes is determined by the order of changing proportions of the LLRs. The node with a larger changing proportion has a greater tendency to be corrected, so it should be preferentially flipped, which ensures the TSBP algorithm keeps a high correctness.

However, we find two problems in the TSBP algorithm. First, the correct results obtained by the previous flipping cannot be used in the subsequent one. Second, since the characteristic of the backtracking algorithm, the correct results in the previous flipping may lead to falling into a new trapping set. To solve the two problems, the OTSBP algorithm is designed. The optimization principle is: when the output state of the second stage encoding is unchanged within some iterations, we pick up two types of target nodes.

In detail, the OTSBP algorithm has five steps as follows.

Step 1. The encoding is implemented by the BP algorithm in the first stage.

Step 2. If all CNs are satisfied, the encoding should be terminated; otherwise, if the preset stop criterion or the maximum iteration are reached, it needs to skip to the next step.

Step 3. Referring to the selection criterion, the two types of target VNs are extracted.

Step 4. The LLR of the target nodes is changed reasonably, and then the BP algorithm is used to encode in the second stage.

Step 5. If the encoding is completed in the second stage, the iteration is terminated; otherwise, the two types of VNs are selected and return to the previous step.

## 4. Simulation Results and Analysis

In this section, the system performance is discussed based on the optimized BP algorithms. The source is the Gaussian sequence following the standard normal distribution N(0,1), and the length is 600. Each simulation compresses 20 blocks of the Gaussian source. The maximum iteration number is 10. In addition, the number of levels in the MLC structure is signified as w=⌈R⌉+1, where *R* is the compression rate, and the code length is equal to the source sequence length.

First, the numbers of unsatisfied nodes are compared with the original BP and the optimized BP algorithms, as shown in Table 1. Here, the P-LDPC code in [17] at the rate of 0.75 is used to simulate the numbers of unsatisfied nodes by implementing these four BP algorithms, respectively. The results are tested under 10 and 20 iterations. It is clear that the improved three algorithms have fewer numbers of unsatisfied nodes after reducing the trapping sets than the original BP algorithm.

**Figure 3:**
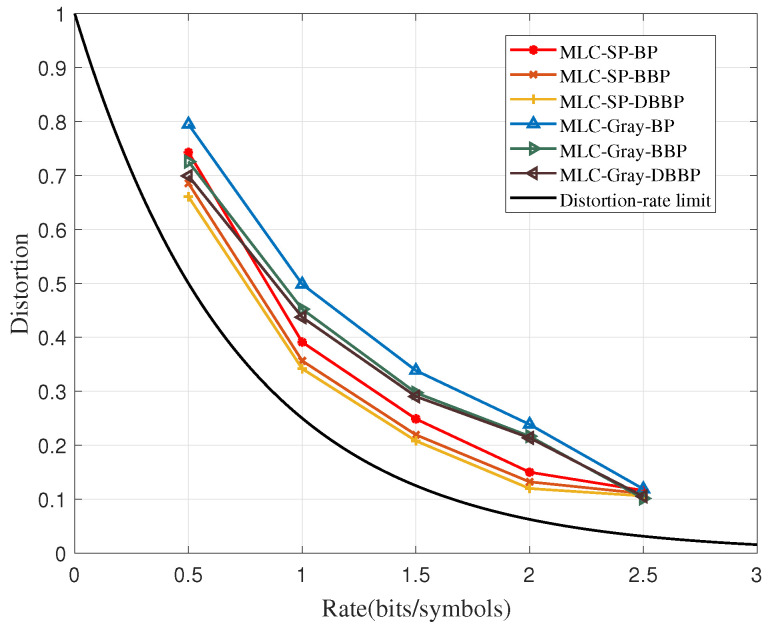
The improvements of the distortion–rate performance obtained by the DBBP algorithms.

Figure 3 compares the distortion–rate performance among the BP, the BBP, and the DBBP algorithms. Two binary mapping methods, including the SP mapping and the Gray mapping, are introduced to verify the advantage of the DBBP algorithm, simultaneously. From Figure 3, both the SP and the Gray mapping methods demonstrate that the DBBP algorithm has the best rate–distortion performance than the BBP and the BP algorithms.

It is found that the coding gains of the BBP are larger than the DBBP algorithms. This confirms the analysis in Section 3.2. Compared to the original BP algorithm, the DBBP algorithm roughly locates the trapping set based on the unsatisfied verification nodes, which greatly reduces the cost of trial and error methods. The DBBP only attempts to modify one node at each time and uses the maximum LLR for verification. In this way, the incorrect probability of the selected nodes is effectively decreased, so that the correct codeword can be obtained by the iteration convergence.

The BBP algorithm can solve most trapping set problems, while the DBBP algorithm only selects the most likely successful backtracking attempts based on the BBP algorithm and carries out backtracking again. In the DBBP algorithm, the second backtracking only aims to solve the new trapping set generated from the last backtracking. When the generation probability of new trapping set is less, the improvement brought by the second backtracking will be not obvious. In this case, the single backtracking algorithm can achieve nearly the same performance with lower complexity.

**Figure 4:**
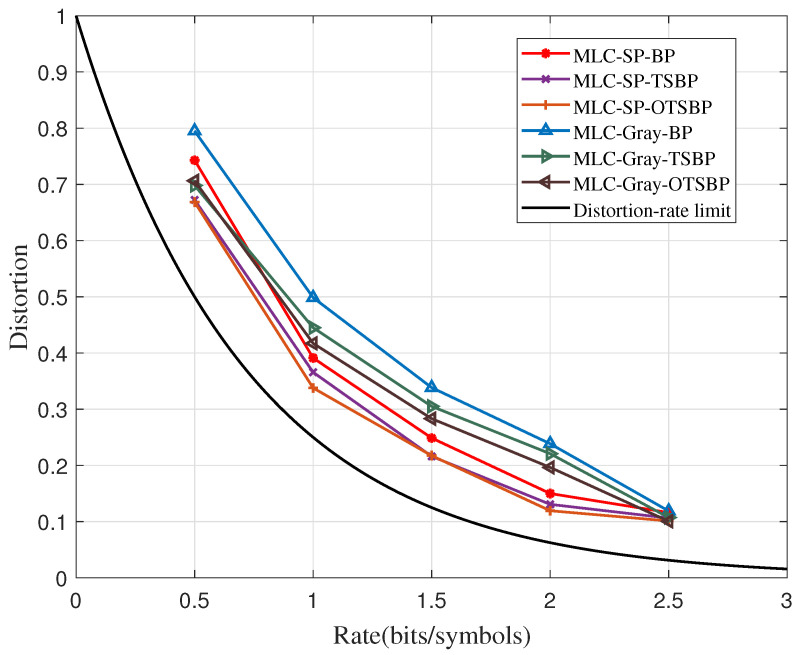
The improvements of the distortion–rate performance obtained by the OTSBP algorithms.

Figure 4 compares the distortion–rate performance of the TSBP and the OTSBP algorithms. The TSBP algorithm selects the abnormal nodes, which are iterated by the BP algorithm, and then it classifies them. The different target nodes are implemented with distinct correction methods; the correct and incorrect information can be amplified and rectified in a reasonable manner. This not only breaks the trapping set, but it also enables a high probability to obtain the correct codeword. Moreover, it accelerates the algorithm convergence to obtain the optimal codeword for source coding. Based on the TSBP algorithm, the correction results at the previous stage are fully utilized in the OTSBP algorithm. Therefore, the new target nodes can be identified, which suppresses the iteration from falling into the new trapping sets.

Some simulation parameters, μ, α, β, and *L* are set as 3, 3, 1.25, and 10, respectively. The maximum iteration number of the two-stage algorithm is 10. In Figure 4, the system distortions based on the TSBP algorithm decrease to 0.0255 and 0.0535 by using the SP and the Gray mapping when R=1, respectively, while the system distortion decrements based on the OTSBP algorithm are 0.0534 and 0.0814. This demonstrates that the OTSBP algorithm has higher effectiveness than the TSBP.

In addition, the TSBP and the OTSBP algorithms can achieve better performance than the BP algorithm by using the Gray mapping when R=0.5. Furthermore, the OTSBP algorithm with the Gray mapping achieves a similar performance in the BP algorithm under the SP mapping at a rate of R=1. Overall, the OTSBP algorithm provides better distortion–rate performance than the other two algorithms at all compression rates by using any mapping methods.

**Figure 5:**
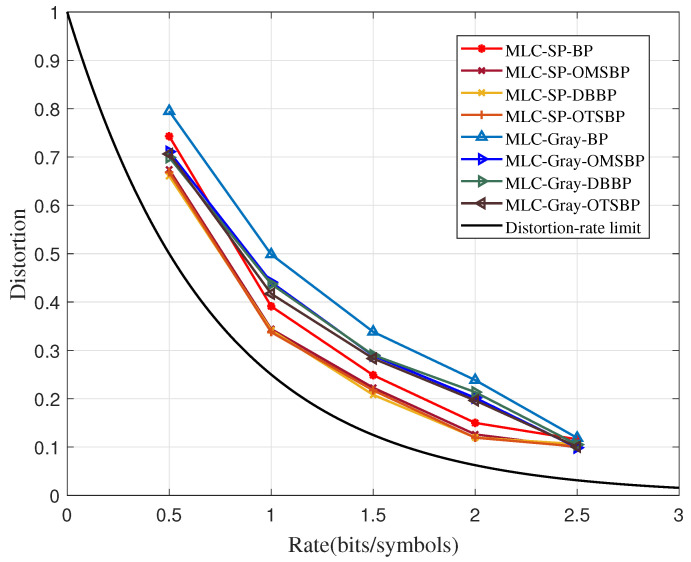
The comparisons of the distortion–rate performance among three optimized BP algorithms.

Figure 5 compares the distortion–rate performance of the OMSBP, the DBBP, and the OTSBP algorithms. The TSBP algorithm classifies the target nodes by selecting the abnormal nodes, which are obtained by the BP algorithm. The correct and incorrect information can be amplified and corrected in a reasonable manner by implementing different correction methods for different target nodes. This not only breaks the trapping set, but it also ensures a high probability of searching the correct codeword with accelerating convergence. The OTSBP algorithm fully utilizes the successful correction results at the previous stage on the basis of the TSBP algorithm. In this case, the iterations will not fall into new trapping sets.

As shown in Figure 5, the OTSBP algorithm achieves the best performance compared to the other two algorithms, followed by the DBBP algorithm, and the OMSBP only outperforms the BP algorithm. Referring to the previous analyses, the DBBP algorithm has higher accuracy and reliability than the MSBP algorithm. The OTSBP algorithm promotes iterations to correctly converge by flipping incorrect nodes and strengthens correct nodes without locating trapping sets. Hence, the OTSBP algorithm is more suitable for realizing the Gaussian source compression than the three algorithms.

## 5. Conclusions

In this paper, three optimal BP algorithms are introduced, namely, the OMSBP, the DBBP, and the OTSBP algorithms, to implement the source coding of the lossy compression system for a Gaussian source. Here, the OMSBP algorithm fully utilizes the amplitude information of suspicious nodes lost by the MSBP algorithm, which avoids selecting target nodes incorrectly. In addition, the OTSBP algorithm employs the error correction attempts at the previous stage of the TSBP algorithm, which can effectively reduce the probability of falling into the new trapping sets at the second stage iteration. Furthermore, the distortion–rate performance demonstrates the improvement derived by the system simulation. Overall, there are two innovations compared to the existing algorithms. First, the negative impact caused by the incorrect selection of target nodes is reduced. Second, the probability of falling into a new trapping set is significantly decreased. The two technical promotions will be useful in improving the related algorithm for achieving system gains.

## Figures and Tables

**Table 1:** The numbers of unsatisfied nodes compared with four different BP algorithms.

Iteration Numbers	BP Algorithm	OMSBP Algorithm	DBBP Algorithm	OTSBP Algorithm
10	36	4	2	0
20	35	3	2	0

## Data Availability

Not applicable.

## References

[1] Van Leemput D., Sabovic A., Hammoud K., Famaey J., Pollin S., De Poorter E. (2023). Energy harvesting for wireless IoT use cases: A generic feasibility model and tradeoff study. IEEE Internet Things J..

[2] Zheng K., Jia X., Chi K., Liu X. (2023). DDPG-Based joint time and energy management in ambient backscatter-assisted hybrid underlay CRNs. IEEE Trans. Commun..

[3] Ma S., Yao H., Mai T., Yang J., He W., Xue K., Guizani M. (2023). Graph convolutional network aided virtual network embedding for Internet of Thing. IEEE Trans. Netw. Sci. Eng..

[4] Wen C., Huang Y., Davidson T.N. (2023). Efficient transceiver design for MIMO dual-function radar-communication systems. IEEE Trans. Signal Process..

[5] Heidari A., Navimipour N.J., Jamali M.A.J., Akbarpour S. (2023). A hybrid approach for latency and battery lifetime optimization in IoT devices through offloading and CNN learning. Sustain. Comput. Inform. Syst..

[6] Berger I., Gibson J. (1998). Lossy source coding. IEEE Trans. Inf. Theory.

[7] Cappellari L. Lossy source compression of non-uniform binary sources using GQ-LDGM codes. Proceedings of the IEEE Information Theory Workshop.

[8] Wainwright M., Maneva E., Martinian E. (2010). Lossy source compression using low-density generator matrix codes: Analysis and algorithms. IEEE Trans. Inf. Theory.

[9] Aref V., Macris N., Urbanke R., Vuffray M. Lossy source coding via spatially coupled LDGM ensembles. Proceedings of the 2012 IEEE International Symposium on Information Theory Proceedings.

[10] Gupta A., Verdu S. Operational duality between lossy compression and channel coding: Channel decoders as lossy compressors. Proceedings of the Information Theory and Applications Workshop.

[11] Golmohammadi A., Mitchell D., Kliewer J., Costello D. (2018). Encoding of spatially coupled LDGM codes for lossy source compression. IEEE Trans. Commun..

[12] Matsunaga Y., Yamamoto H. (2003). A coding theorem for lossy data compression by LDPC codes. IEEE Trans. Inf. Theory.

[13] Chen P., Shi L., Fang Y., Lau F.C.M., Cheng J. (2023). Rate-diverse multiple access over Gaussian channels. IEEE Trans. Wire. Commun..

[14] Fang Y., Zhuo J., Ma H., Mumtaz S., Li Y. (2023). Design and analysis of a new index-modulation-aided DCSK system with frequency-and-time resources. IEEE Trans. Veh. Technol..

[15] Divsalar D., Dolinar S., Jones C.R., Andrews K. (2003). Capacity-approaching protograph codes. IEEE J. Select. Areas Commun..

[16] Song D., Ren J., Wang L., Chen G. (2023). Gaussian source coding based on P-LDPC code. IEEE Trans. Commun..

[17] Deng H., Song D., Miao M., Wang L. Design of lossy compression of the Gaussian source with protograph LDPC codes. Proceedings of the 2021 15th International Conference on Signal Processing and Communication Systems (ICSPCS).

[18] Ren J., Song D., Wu H., Wang L. (2023). Lossy P-LDPC codes for compressing general sources using neural networks. Entropy.

[19] Filler T., Fridrich J. (2007). Binary quantization using belief propagation with decimation over factor graphs of LDGM codes. arXiv.

[20] Braunstein A., Kayhan F., Zecchina R. Efficient LDPC codes over GF(q) for lossy data compression. Proceedings of the 2009 IEEE International Symposium on Information Theory.

[21] Honda J., Yamamoto H. (2014). Variable length lossy coding using an LDPC code. IEEE Trans. Inf. Theory.

[22] Liu Y., Wang L., Wu H., Liu S. Performance of lossy P-LDPC codes over GF(2). Proceedings of the International Conference on Signal Processing and Communication Systems.

[23] Fang Y., Zhang G., Cai G., Lau F.C.M., Chen P., Han G. (2019). Root-protograph-based BICM-ID: A reliable and efficient transmission solution for block-fading channels. IEEE Trans. Commun..

[24] Chen P., Xie Z., Fang Y., Chen Z., Mumtaz S., Rodrigues J.J.P.C. (2020). Physical-layer network coding: An efficient technique for wireless communications. IEEE Netw..

[25] Song D., Wang L., Chen p. (2023). Mesh model-based merging method for DP-LDPC code pair. IEEE Trans. Commun..

[26] Song D., Wang L., Xu Z., Chen G. (2022). Joint code rate compatible design of DP-LDPC code pairs for joint source channel coding over implant-to-external channel. IEEE Trans. Wirel. Commun..

[27] Song D., Ren J., Wang L., Chen G. (2022). Designing a common DP-LDPC code pair for variable on-body channels. IEEE Trans. Wirel. Commun..

[28] Dai L., Fang Y., Guan Y.L., Guizani M. (2023). Design of protograph LDPC-coded MIMO-VLC systems with generalized spatial modulation. China Commun..

[29] Zhang X., Chen S. (2015). A two-stage decoding algorithm to lower the error-floors for LDPC codes. IEEE Commun. Lett..

[30] Amiri B., Kliewer J., Dolecek L. (2015). Analysis and enumeration of absorbing sets for non-binary graph-based codes. IEEE Trans. Commun..

[31] Martinia E., Yedidia J. (2004). Iterative quantization using codes on graphs. arXiv.

[32] Aref V., Macris N., Vuffray M. (2015). Approaching the rate-distortion limit with spatial coupling, belief propagation, and decimation. IEEE Trans. Inf. Theory.

[33] Miyake S., Muramatsu J. A coding theorem for lossy data compression by LDPC codes. Proceedings of the IEEE International Symposium on Information Theory.

[34] Han Y., Ryan W. (2015). Low-floor decoders for LDPC codes. IEEE Trans. Commun..

[35] Shin B., Park H., No J., Chung H. (2011). Multi-stage decoding scheme with post-processing for LDPC codes to lower the error floors. IEICE Trans. Commun..

[36] Kang J., Huang Q., Lin S., Abdel-Ghaffar K. (2011). An iterative decoding algorithm with backtracking to lower the error-floors of LDPC codes. IEEE Trans. Commun..

